# IL-27 Inhibits Anti-*Mycobacterium tuberculosis* Innate Immune Activity of Primary Human Macrophages

**DOI:** 10.1016/j.tube.2023.102326

**Published:** 2023-02-24

**Authors:** Hailey Gollnick, Jamie Barber, Robert J Wilkinson, Sandra Newton, Ankita Garg

**Affiliations:** 1 Department of Infectious Diseases, College of Veterinary Medicine, University of Georgia, Athens, GA, USA; 2 College of Veterinary Medicine, University of Georgia, Athens, GA, USA; 3 Department of Infectious Diseases, Imperial College London, W12 0NN, United Kingdom; 4 The Francis Crick Institute London NW1 1AT, United Kingdom; 5 Section of Paediatric Infectious Disease, Department of Infectious Disease, Imperial College London, United Kingdom, W2 1PG

**Keywords:** IL-27_1_, *M. tuberculosis*_2_, human macrophages_3_, innate immunity_4_, cytokiness_5_

## Abstract

*Mycobacterium tuberculosis* (*M tuberculosis*) is an intracellular pathogen that primarily infects macrophages. Despite a robust anti-mycobacterial response, many times macrophages are unable to control *M. tuberculosis*. The purpose of this study was to investigate the mechanism by which the immunoregulatory cytokine IL-27 inhibits the anti-mycobacterial activity of primary human macrophages. We found concerted production of IL-27 and anti-mycobacterial cytokines by *M. tuberculosis-infected* macrophages in a toll-like receptor (TLR) dependent manner. Notably, IL-27 suppressed the production of anti-mycobacterial cytokines TNFα, IL-6, IL-1β, and IL-15 by *M. tuberculosis*-infected macrophages. IL-27 limits the anti-mycobacterial activity of macrophages by reducing Cyp27B, cathelicidin (LL-37), LC3B lipidation, and increasing IL-10 production. Furthermore, neutralizing both IL-27 and IL-10 increased the expression of proteins involved in LC3-associated phagocytosis (LAP) pathway for bacterial clearance, namely vacuolar-ATPase, NOX2, and RUN-domain containing protein RUBCN. These results implicate IL-27 is a prominent cytokine that impedes *M. tuberculosis* clearance.

## 1 Introduction

*Mycobacterium tuberculosis* (*M. tuberculosis*), the causative agent for tuberculosis is a highly successful intracellular pathogen making it one of the leading infectious causes of mortality worldwide ^1^. *M. tuberculosis* resides in the macrophages and manipulates macrophage-induced host cell defense mechanisms such as antigen presentation, phagosomal maturation, cytokine production, and antimicrobial pathways for its own survival ^2, 3^. The engagement of surface Toll-like receptors (TLR)-2 and -4 on macrophages with its cognate *M. tuberculosis* pathogen-associated molecular pattern (PAMP) activates signaling and phagolysosome pathways important for the production of cytokine/chemokines and *M. tuberculosis* degradation, respectively ^4–7^. TLR-2 and -4 mediate the release of cytokines TNFα, IL-6, IL-1β, IL-12 by macrophages in response to *M. tuberculosis* infection, which is critical for host resistance to infection due to their ability to activate cathelicidin (hCAP) and defensin mediated direct antimicrobial mechanisms ^8–12^ However, despite strong anti-*M. tuberculosis* innate immune mechanisms a substantial proportion of individuals infected with *M. tuberculosis* are unable to control the infection.

IL-27 is a heterodimeric cytokine of the IL-12 family, composed of p28 and Epstein-Barr virus-induced protein 3 (EBI3) subunits that are structurally similar to the p35 and p40 subunits of IL-12, respectively ^13^. Activated macrophages and dendritic cells secrete IL-27, which regulates both innate and adaptive immune responses. Unlike IL-12, IL-27 inhibits anti-*M. tuberculosis* T cell effector functions, and phagosomal acidification and compromises control of *M. tuberculosis* growth by macrophages ^14–17^ An increased level of IL-27 has been reported during *M. tuberculosis* infection and treatment with IL-12 along with IL-27 neutralization restricts the growth of *M. tuberculosis* ^15, 18^. We recently reported that neutralizing IL-27 controls replication of *M. tuberculosis* in the settings of HIV-*M. tuberculosis* co-infection ^19^. The underlying mechanism by which IL-27 impedes anti-mycobactericidal activity of macrophages remains less defined.

The purpose of the present study was to examine the effect of IL-27 on host defense of primary human macrophages *in vitro* infected with *M. tuberculosis*. We present evidence that IL-27 is produced by human macrophages in TLR dependent manner along with other anti-*M. tuberculosis* cytokines. We further show that IL-27 inhibits proinflammatory cytokine production in response to infection with *M. tuberculosis*, and subsequent mycobactericidal activity of macrophages. These results provide an insight into the immune therapeutic approaches that may be adopted to control *M. tuberculosis* infection.

## 2 Materials and Methods

### 2.1 Patient population

This study was reviewed and approved by the Institutional Review Board of the University of Georgia, Athens State IRB reference if available. The donors were healthy, HIV seronegative and QuantiFERON negative adults who provided written informed consent prior to donating blood.

### 2.2 Cell isolation and generation of primary human macrophages

Peripheral blood mononuclear cells (PBMC) were isolated from freshly obtained blood by Ficoll density centrifugation (GE Healthcare) and used for the generation of primary monocyte-derived macrophages (hereafter macrophages). Monocytes were isolated by plate adherence method, and subsequently cultured with RPMI1640 supplemented with AB human serum and M-CSF (10 ng/ml) for 7-days. Media was replaced every third day with fresh culture media supplemented with M-CSF.

### 2.3 *M. tuberculosis* culture

*M. tuberculosis* Erdman, provided by Dr. Fred Quinn (University of Georgia, Athens), was maintained in Middlebrook 7H9 broth containing albumin, dextrose, catalase (ADC) as previously described ^20^. For macrophages infections, bacteria were plated on Middlebrook 7H10 agar containing 10% oleic acid-albumin-dextrose-catalase (OADC). All assays involving *M. tuberculosis* were performed in Biosafety Level-3 following the institutional biosafety guidelines.

### 2.4 Macrophage infection and treatment

Macrophages (0.3 x 10^6^ / well) were plated in 12-well plates in antibiotic-free RPMI 1640 medium and 10% human serum. Cells were infected with Erdman at a multiplicity of infection (MOI) of 1:5 for 3 hrs; subsequently, cells were washed and treated with Gentamycin Sulfate (30 μg/ml; VWR Life Sciences) for additional 2 hours to kill extracellular bacteria, and cultured in RPMI 1640 with 10% human serum. For some experiments, macrophages were incubated with blocking anti-TLR2 or -TLR4 or isotype-matched control antibodies (10 μg/ml) (all from Thermo Scientific) for 30 minutes at 37°C followed by infection with *M. tuberculosis*. For some experiments, *M. tuberculosis*-infected macrophages were cultured with recombinant (r) - IL-27 (10 or 15 ng/ml), neutralizing IL-27 or isotype matched control antibodies (all from R&D systems), IL-10 (Thermo Scientific) antibodies (10 μg/ml), Rapamycin (200 nM) or Bafilomycin A (200 nM). For some experiments, macrophages were infected with *M. tuberculosis* strain lacking 19 kDa lipoprotein (Δ19kDa), or *M. tuberculosis* strain complemented for 19 kDa (19::19)^21^. The derivation and characterization of these strains has previously been described Culture supernatants or cellular lysates were prepared at 24-hours post-infection and stored at -80°C until further use.

To determine the effect of protein phosphatase 2ac (PP2ac), *M. tuberculosis*-infected macrophages were cultured with PP2Ac agonist forskolin (FK) or antagonist okadaic acid (OA) (both from Tocris), intracellular replication or expression of proteins was measured in the cellular lysates at 24-hours post-infection.

### 2.5 Measurement of cell cytotoxicity

Cell cytotoxicity of macrophages infected with *M. tuberculosis* and treated with rIL-27, or anti-IL-27 and IL-10 antibodies was determined using CyQUANT™ LDH cytotoxicity assay kit according to manufacturer’s instructions (Supplementary Figure 1). Briefly, 50 μl of culture supernatant was added to equal volume of reaction mixture and incubated at room temperature for 30 minutes, followed by addition of stop solution. Absorbance was measured at 490nm and 680 nm as reference wavelength. Cells treated with lysis buffer, and untreated media served as maximum LDH and spontaneous LDH activity controls, respectively; % cytotoxicity = [Treatment LDH activity – spontaneous LDH activity / maximum LDH activity – spontaneous LDH activity] x 100. Data is presented as percentage viability = 100 - % cytotoxicity.

### 2.6 Measurement of intracellular *M. tuberculosis*

Macrophages were infected with *M. tuberculosis* as detailed above. Cells were lysed with 0.1% SDS at day-0 and day -3 post-infection, and cellular lysates were serially diluted and plated in triplicate on Middlebrook 7H10 agar supplemented with OADC enrichment. The number of colonies was counted after 3 weeks and colony-forming units (CFU) / ml was determined.

### 2.7 Flow cytometry

For immune phenotyping, macrophages were immunolabeled with anti-human-CD14-PE/Cy7, -CD11b-APC-eFlour780, -CD36-Alexa Flour488, -CD169-PE/Cy7, -CD206-PE/Dazzle594, -CX3CR1-PE, -HLA DR-eFlour450 and –CD68-PE antibodies. Controls for each experiment included unstained cells and fluorescence minus one (FMO). Cells were collected on Quanteon and results were analyzed with FlowJo software. A minimum of 20, 000 gated events were collected for each sample.

### 2.8 Fluorescence microscopy

Macrophages were infected with *M. tuberculosis* as detailed above. Cells were fixed for 30 minutes using formaldehyde, then permeablized with 0.2% Triton X-100 for 10 minutes, and incubated with 2% bovine serum albumin to block non-specific binding of antibodies. Cells were incubated with anti-human-p62-Alexa Fluor 647 or –NOX2-APC/Cy7 at 1:100 dilution overnight at 4°C, followed by mounting with Prolong Gold antifade agent with nuclear stain DAPI. Cells were imaged on Nikon A1R confocal microscope using 75X objective.

### 2.9 Immunoblotting

Immunoblotting of cellular lysates (35 μg) was performed as previously described ^22^. Relative densities for target protein bands Cyp27B1 (57 kDa), PP2Ac (36 kDa), LC3B-II (14 kDa), p62 (62 kDa), NOX2/gp91^phox^ (65 kDa), ATPase (75 kDa) and RUBCN (140 kDa) were normalized to housekeeping β-actin (45 kDa) or GAPDH (37 kDa) bands compared using ImageJ (NIH). Normalized ratiometric data were log2 transformed.

### 2.10 Quantitative Reverse-Transcription Polymerase chain reaction (qRT-PCR)

qRT-PCR was performed as previously described ^19^. Total RNA was isolated from macrophages using TRIzol™ reagent (Thermo Fisher Scientific) according to the manufacturer’s protocol; 500 ng RNA was used for cDNA synthesis using Superscript II reverse transcriptase (Thermo Fisher Scientific). SyBrGreen master mix (Thermo Fisher Scientific) was used in a 10μL reaction volume that included 10 ng of cDNA for primer pairs (1 μM) specific for Human Cytochrome P450 oxidase (Cyp7B1; forward CACCCGACACGGAGACCTT, reverse TCAACAGCGTGGACACAAACA). The Human acidic ribosomal protein (HuPO; forward CATTCTATCATCAACGGGTACAA, reverse AGCAAGTGGGAAGGTGTAATCC) was used as a housekeeping gene. Data were analyzed to calculate the relative quantification of the Cyp27B1 gene in comparison to the HuPO gene by comparative Ct method (2-ΔCt) ^23^.

### 2.11 Quantification of cytokines and hCAP

Supernatants collected and stored at -80°C were used to determine levels of TNFα, IL-6, IL-1β, and IL-10 by ELISA (Biolegend, San Diego); IL-27 was measured using Duoset ELISA kit (R&D Systems). Net cytokine production = Cytokine produced by *M. tuberculosis-infected* macrophages – cytokine produced by uninfected controls. To determine the effect of IL-27 on cytokine production, IL-12p70, TNFα, IL-6, IL-23, IL-8, IL-18, IL-15, and IL-1β were determined in the culture supernatants at 24 hrs post-infection using LUMINEX multiplex system (R&D Systems) and custom-designed kit.

The quantity of hCAP was measured in the culture supernatants at 24 hrs post-infection using hCAP LL-37 kit (Hycult Biotech).

### 2.12 Statistical analysis

Data are expressed as mean values ± standard error mean (SEM). Paired Student’s t-tests were used to determine the statistical significance for *in vitro* experiments. Statistical analysis was performed using Graphpad Prism 8 (La Jolla, CA). P-values of <0.05 were considered statistically significant.

## 3 Results

### 3.1 Concurrent production of IL-27 and proinflammatory cytokines by human macrophages

**Figure 1:**
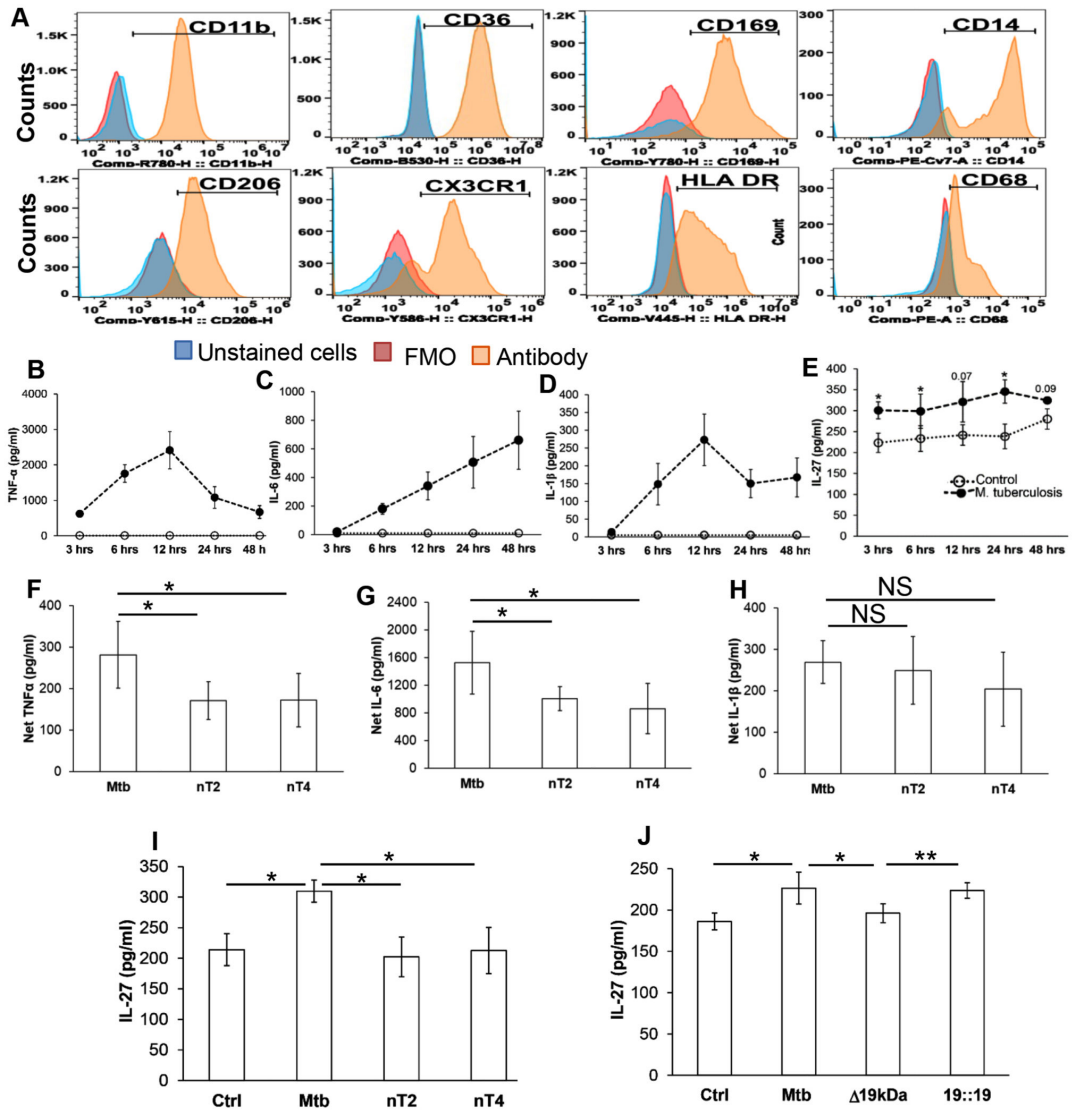
Concurrent production of proinflammatory cytokines and IL-27 by primary human macrophages in response to infection with *M. tuberculosis* Monocytes from PBMC from HIV (-) Quantiferon (-) healthy participants were isolated by plate adherence, and cultured in presence of M CSF for 7-days to generate macrophages. **(A)** Cells were surface stained with anti-CD11b, -CD36, - CD169, -CD14, -CD206, -HLA DR and CX3CR1 antibodies; cells were fixed / permeablized and stained with anti-CD68. The expression of surface markers and intracellular CD68 was measured by flow cytometry**. (B-E)** Cells were infected with *M. tuberculosis* at an MOI of 1:5 for 3 hours, washed with PBS to remove extracellular bacteria. The quantity of cytokines was measured at indicated time points in the culture supernatants. **(F-I)** Cells were incubated with isotype or anti-TLR-2 or TLR-4 antibodies (10 μg/ml) for 30 minutes prior to infection with *M. tuberculosis*. The quantity of cytokines was measured in the culture supernatants at 24-48 hours. **(J)** Cells were infected with *M. tuberculosis, M. tuberculosis* lacking 19 kDa lipoprotein (Δ19kDa) or the recombinant *M. tuberculosis* strain complemented by 19 kDa lipoprotein (19::19). The quantity of cytokine was measured in the culture supernatants at 24-48 hours. For (A) Representative flow cytometer histogram plot is shown of N=5 donors. FMO; Fluorescence minus one. For (F-H) Net cytokines production was calculated as quantity in presence of *M. tuberculosis* - Quantity in absence of *M. tuberculosis*. Data shown are for N=4 donors; mean values +/- SEM are shown. *p<0.05, NS: Non-significant

*M. tuberculosis*-infected macrophages elicit cytokine production that plays a critical role in deciding the outcome of *M. tuberculosis* infection. Among these TNF-α, IL-6 and IL-1β are critical for an optimal innate immune-mediated control of *M. tuberculosis*. Initially, we characterized our primary human macrophage system by flow cytometry. Our primary human macrophages expressed CX3CR1 similar to lung “tissue-resident” macrophages and consisted of CD11b^+^HLA DR^+^CD169^+^CD206^+^ macrophages (alveolar macrophages) and HLA DR^+^CD14^+^CD36^+^ macrophages (interstitial macrophages) (Figure 1A), thus reflecting the similarities with lung resident cells ^24–26^. Subsequently, we infected primary human macrophages with *M. tuberculosis*; similar to previous studies we found *M. tuberculosis*-infected macrophages produced pro-inflammatory cytokines as early as 3-hours post-infection (Figure 1B-D). IL-27 is an immune-regulatory cytokine that has both proinflammatory and anti-inflammatory properties, and its manipulation influences proinflammatory cytokine production by macrophages. We found human macrophages also produce IL-27 upon infection with *M. tuberculosis* (Figure 1E and Supplementary Figure 2).

Signaling pathways activated because of engagement of TLRs with its cognate *M. tuberculosis* pathogen-associated molecular pattern (PAMP) are important for cytokine production by macrophages ^2, 4, 5^. In this regard, we found that blocking TLR-2 or -4 prior to infection with *M. tuberculosis* decreased the production of TNF-α and IL-6 (Figure 1F-G). Of note, similar to TNF-α and IL-6, the quantity of IL-27 decreased when TLR-2 or TLR-4 were blocked prior to infection (Figure 1I and Supplementary Figure 2). Furthermore, infection with *M. tuberculosis* strain lacking 19 kDa lipoprotein, putative *M. tuberculosis* TLR-2 ligand, produced less IL-27 as compared to wild type *M. tuberculosis* or *M. tuberculosis* complemented with the 19 kDa lipoprotein (Figure 1J). Collectively these studies demonstrate that *M. tuberculosis*-induced TLR signaling not only activates macrophages to produce anti-*M. tuberculosis* cytokines, but also induces immunoregulatory cytokines such as IL-27 in a TLR dependent manner.

### 3.2 IL27 and intracellular replication of *M. tuberculosis*

**Figure 2:**
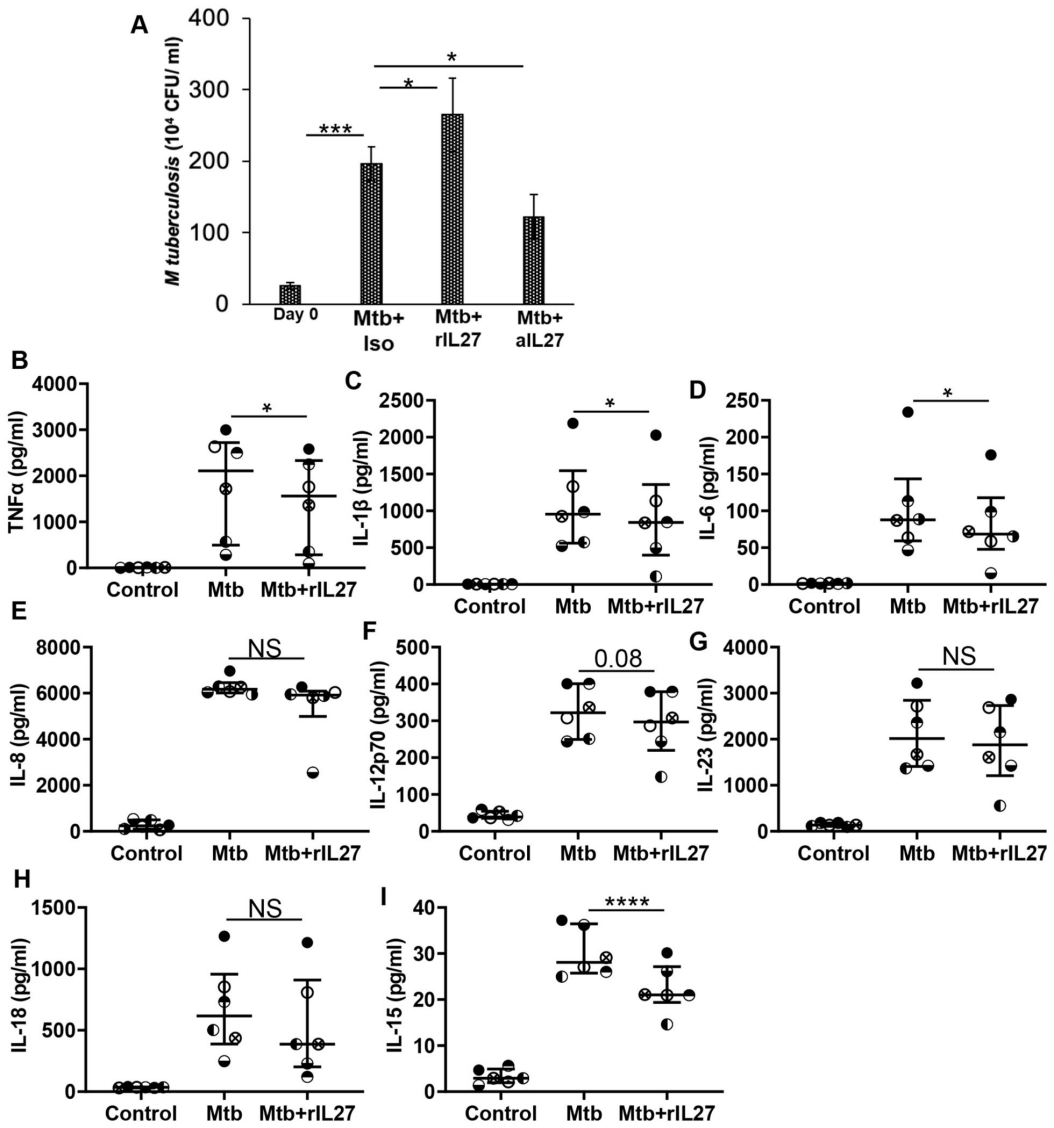
IL-27 regulates intracellular *M. tuberculosis* growth and innate cytokine production by macrophages Cells were infected with *M. tuberculosis* at an MOI of 1:5 for 3 hours, washed with PBS to remove extracellular bacteria **(A)** *M. tuberculosis* growth (colony forming units (CFU) / ml) was determined in the cellular lysates at day-0 post-infection. Infected cells were treated with isotype (Mtb+Iso) of neutralizing IL-27 (Mtb+aIL27) antibody or rIL27 (Mtb+rIL27) and CFU/ml was determined at day-3 post-infection. **(B-I)** *M. tuberculosis-infected* cells were treated with rIL-27 (10 ng/ml), amount of cytokines was measured in the culture supernatants of uninfected (Control), *M. tuberculosis-infected* (Mtb), and *M. tuberculosis-infected* treated with rIL-27 (Mtb +rIL27) cells at 24-48 hours post-infection. Histograms shown are for (A) N=9 donors. Histograms show mean values +/- SEM. (B-I) Each dot in the plots depicts data of each individual donor, the plots include observations from 25^th^ to 75^th^ percentile. The horizontal line represents the median value. *p<0.05, ***p<0.0005, ****p<0.00005.

Jung *et al* demonstrated IL-27 inhibits vacuolar ATPase to downregulate the bactericidal activity of macrophages for Gram (+) and Gram (−) bacteria, and *M. tuberculosis* ^15, 16^. In the next set of experiments, we sought to determine the effect of IL-27 on the intracellular replication of *M. tuberculosis*. For this, macrophages were infected with *M. tuberculosis* at an MOI 1:5 for 3 hours and subsequently treated with neutralizing IL-27 antibody or rIL-27. Intracellular replication of *M. tuberculosis* was determined at day-3 post-infection. We found increased mycobacterial counts in the cellular lysates at day-3 post-infection compared to day-0 cellular lysates (26 ± 4.3 x 10^4^ vs 197 ± 23.4 x 10^4^ CFU/ml; p=0.0003). *M. tuberculosis* counts declined in lysates of macrophages cultured with anti-IL-27 antibody (197 ± 23.4 x 10^4^ vs 122 ± 30 x 10^4^ CFU/ml; p=0.05), and increased in macrophages cultured with rIL-27 (197 ± 23.4 x 10^4^ vs 265 ± 50 x 10^4^ CFU/ml; p=0.05) (Figure 2A).

Subsequently, we determined the effect of IL-27 on anti-mycobacterial cytokines produced by macrophages. For these studies, we measured the cytokines in the culture supernatants of macrophages infected with *M. tuberculosis* and subsequently cultured with rIL-27 for 24 hours. We found increased quantities of TNF-α, IL-6, IL-1β, IL-8, IL-12p70, IL-23, IL-18 and IL-15 in the culture supernatants of *M. tuberculosis*-infected macrophages as compared to uninfected controls. However, the addition of rIL-27 inhibited the production of TNF-α, IL-6, IL-1β, and IL-15, but did not affect other cytokines (Figure 2B-I). These studies show that IL-27 inhibits anti-mycobacterial cytokine production, and augments intracellular replication of *M. tuberculosis*.

### 3.3 IL-27 inhibits the mycobactericidal activity of human macrophages

**Figure 3:**
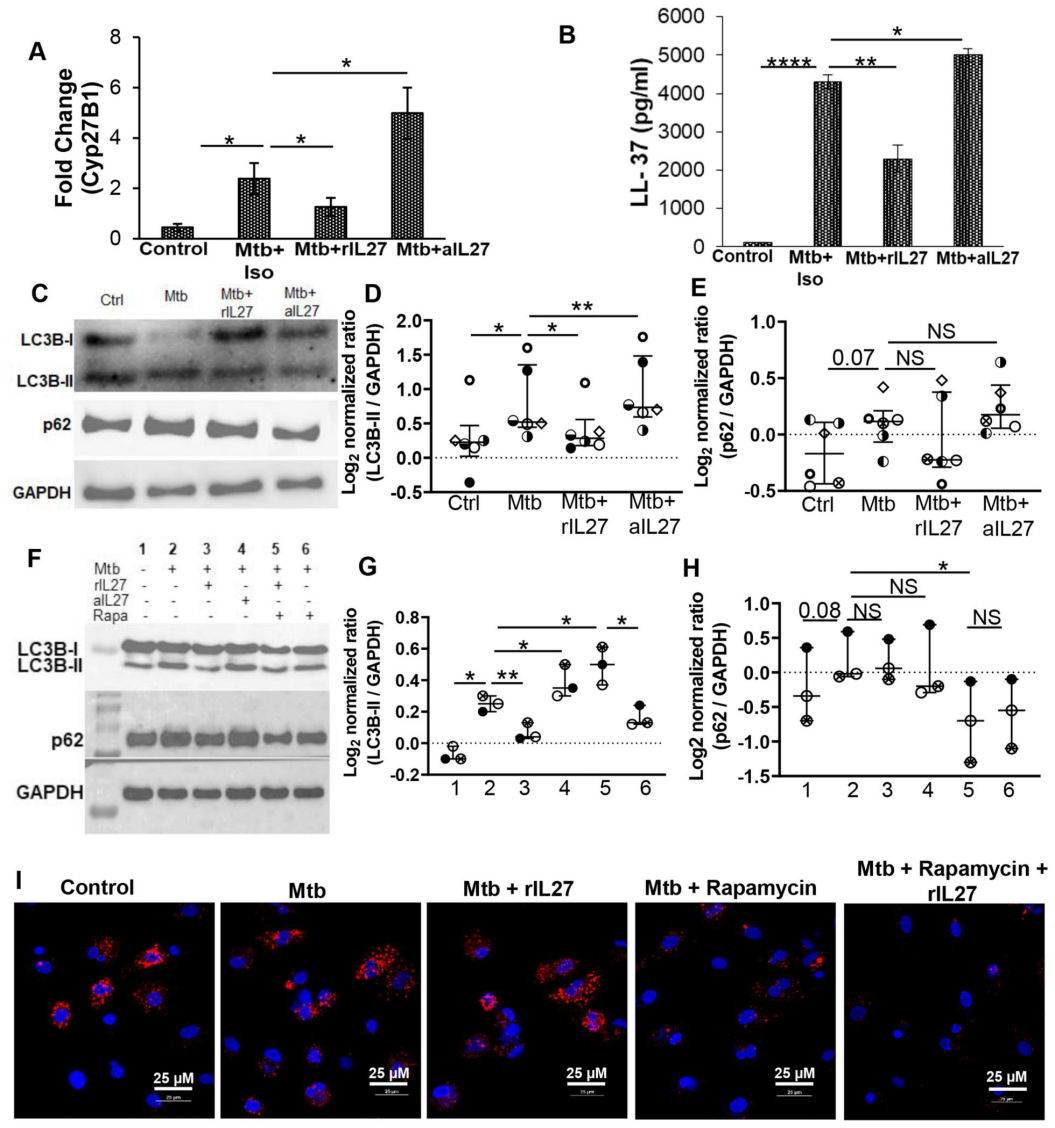
IL-27 and anti-mycobacterial activity of macrophages Cells were uninfected (Control) or infected with *M. tuberculosis* at an MOI of 1:5 for 3 hours, washed with PBS to remove extracellular bacteria and subsequently treated with isotype (Mtb+Iso) or neutralizing IL-27 (Mtb+aIL27) antibodies, or rIL27 (Mtb+rIL27) for 24-48 hours **(A)** expression of housekeeping gene HuPO and Cyp27B1 was determined using SYBR green, and fold change was calculated **(B)** Amount of LL-37 was measured in the culture supernatants **(C-H)** Total cellular lysates were prepared and immunoblotted using anti-GAPDH (1:4000), - LC3B (1:1000) and – p62 (1:5000) antibody. A representative immunoblots are shown shown. For (A) and (B), the histogram shown is for N=6 donors; histograms show mean values +/- SEM. (D-E and G-H) Each dot in the plots depicts data of each individual donor, the plots include observations from 25^th^ to 75^th^ percentile. The horizontal line represents the median value. **(I)** Uninfected (Control) or *M. tuberculosis* infected (Mtb) cells were cultured in the presence or absence of indicated treatment, and IF was performed with anti-p62 (red) antibody, and nuclei were stained with DAPI (blue). Image is representative of N=3 donors. *p<0.05, **p<0.005, ****p<0.00005.

We found IL-27 inhibits *M. tuberculosis-induced* IL-15 production (31 ± 2 vs 22 ± 2.1 pg/ml; p= 0.0003) (Figure 2I). IL-15 is important to maintain memory T cells, and for the induction of Cyp27B1 and the downstream antimicrobial peptide hCAP critical for the anti-mycobacterial activity of macrophages ^10, 27^. In this study, we sought to determine if IL-27 modulates Cyp27B1 and hCAP levels in response to infection with *M. tuberculosis*. For this, *M. tuberculosis-infected* macrophages were cultured with rIL-27 or anti-IL-27 antibody, and gene expression of Cyp27B1 and quantity of hCAP was determined in the cellular lysates and culture supernatants, respectively. Compared to uninfected controls, macrophages infected with *M. tuberculosis* exhibited 2.4 fold increased expression of Cyp27B1 (0.5 ± 0.15 vs 2.4 ± 0.6; p=0.02). Addition of rIL-27 decreased, and neutralizing IL-27 increased Cyp27B1 expression, respectively (2.4 ± 0.6 vs 1.2 ± 0.36; p=0.02, and 2.4 ± 0.6 vs 4.98 ± 1.01; p=0.04, respectively) (Figure 3A).

Concomitant to Cyp27B1 expression, the quantity of hCAP was more in culture supernatants of macrophages infected with *M. tuberculosis* (100 vs 4300 ± 192 pg/ml; p=0.0005); rIL-27 decreased hCAP in dose-dependent manner with significant reduction at 10 ng/ml (4300 ± 192 vs 2296 ± 361 pg/ml; p=0.007) (Figure 3B and Supplementary Figure 3A). Of note, neutralizing IL-27 increased the quantity of hCAP produced by *M. tuberculosis-infected* macrophages (4300 ± 192 vs 5000 ± 164 pg/ml; p=0.01) (Figure 3B). Collectively, these findings suggest that IL-27 inhibits the antimicrobial activity of macrophages.

It has previously been demonstrated that hCAP mediates the anti-mycobacterial activity of macrophages by upregulation of the autophagy pathway, which results in the formation of autophagosomes ^9, 28^. In the next set of experiments, we sought to study if IL-27 interferes with the autophagy pathway of macrophages infected with *M. tuberculosis*. During autophagy, cytosolic microtubule-associated protein 1 light chain 3B (LC3B)-I is converted to LC3B-II (LC3B lipidation); increased expression of LC3B-II is an indicator of autophagy induction, or its accumulation due to the inhibition of autophagic flux (degradation of polyubiquitin-binding protein p62 (sequestosome 1). Initially, we studied autophagy in macrophages infected with *M. tuberculosis*. Consistent with previous findings, we found infection with *M. tuberculosis* increased LC3B lipidation but inhibited p62 degradation. Importantly, treatment with rapamycin induced autophagic flux (Supplementary Figure 3B). Subsequently, we studied the effect of IL-27 on autophagy induction by measuring the expression of LC3B-II in the cellular lysates of macrophages infected with *M. tuberculosis* and cultured in the presence of rIL-27 or neutralizing IL-27 antibody. The expression of LC3B-II was more in *M. tuberculosis-infected* macrophages as compared to uninfected controls (0.24 ± 0.15 vs 0.66 ± 0.2; p=0.04); addition of rIL-27 decreased whereas neutralizing IL-27 increased LC3B-II expression (0.66 ± 0.2 vs 0.35 ± 0.1; p=0.04 and 0.66 ± 0.2 vs 0.8 ± 0.2; p=0.001, respectively) (Figure 3C and D). Furthermore, compared to *M. tuberculosis* infection, treatment of *M. tuberculosis* infected macrophages with rapamycin increased LC3B-II expression (0.66 ± 0.2 vs 0.04 ± 0.1; p=0.01), addition of rIL-27 decreased its expression (0.66 ± 0.2 vs 0.04 ± 0.1; p=0.01) (Figure 3F and G). Subsequently, we determined the effect of IL-27 on p62 degradation. As compared to uninfected controls, *M. tuberculosis* infection resulted in the accumulation of p62 (-0.14 ± 0.1 vs 0.15 ± 0.25; p=0.03) indicating the inhibition of autophagic flux; addition of rIL-27 or neutralizing IL-27 did not affect p62 expression (0.15 ± 0.25 vs 0.06 ± 0.41; p=0.1 and 0.15 ± 0.25 vs 0.21 ± 0.1; p=0.56, respectively) (Figure 3C and E). Of note, compared to *M. tuberculosis* infection, treatment of *M. tuberculosis* infected macrophages with rapamycin decreased p62 levels (0.15 ± 0.25 vs 0.04 ± 0.1; p=0.01). Notably, addition of rIL-27 did not affect p62 expression (0.3 ± 0.07 vs 0.04 ± 0.1; p=0.01) (Figure 3F and H). Immunofluorescence also showed few p62 puncta in *M. tuberculosis* infected macrophages treated with rapamycin, the addition of rIL27 did not affect rapamycin induced p62 puncta (Figure 3I). A previous study demonstrated that inhibition of serine/threonine protein phosphatase-2Ac (PP2Ac) increased LC3B-II and decreased p62 expression in *M. bovis* infected mouse macrophages. We found that the inhibition in the expression of LC3B-II by IL-27 was independent of serine/threonine protein phosphatase-2Ac (PP2Ac) (Supplementary Figure 4). Of note, treatment with PP2Ac inhibitor OA decreased intracellular replication of *M. tuberculosis* (Supplementary Figure 4B). Collectively, these findings suggest that IL-27 inhibits the anti-mycobacterial activity of macrophages by inhibiting autophagy induction.

### 3.4 IL-27 regulates the expression of proteins involved in LC3 associated phagocytosis (LAP)

**Figure 4:**
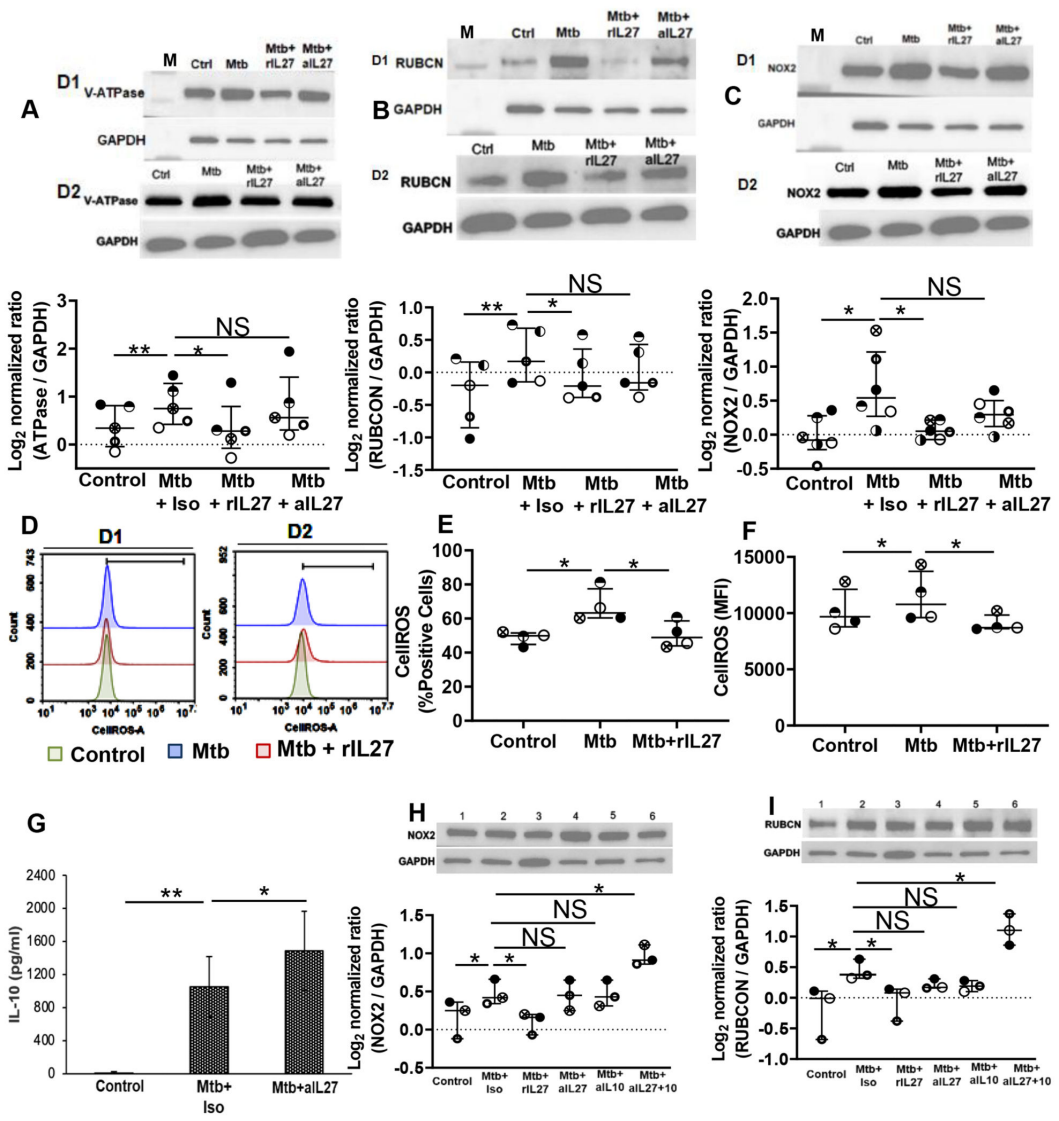
IL-27 regulates the expression of LAP pathway in response to *M. tuberculosis* Cells were uninfected (Ctrl) of infected with *M. tuberculosis* at MOI 1:5 for 3 hours, washed with PBS to remove extracellular bacteria and **(A-C)** treated with isotype (Mtb+Iso) or neutralizing IL-27 (Mtb+IL27) antibodies or rIL27 (Mtb+rIL27). Cellular lysates were prepared at 24-48 hours post-infection and immunoblotted using anti-GAPDH, -vacuolar ATPase (V-ATPase), - Rubicon (RUBCN) and – NOX2 antibody. Representative immunoblot of two donors (D1 and D2) are shown. M; molecular weight marker **(D-F)** ROS was measured by flow cytometry. Representative flow cytometry plots for two donors (D1 and D2) are shown. **(G)** The Amount of IL-10 was measured in the culture supernatants at 24-48 hours post-infection. **(H-I)** In addition to rIL27 and neutralizing IL-27, *M. tuberculosis-infected* cells were treated with neutralizing IL-10 or neutralizing IL-27+IL-10 antibodies. Cellular lysates were prepared at 24-48 hours post-infection and immunoblotted using anti-GAPDH, - Rubicon (RUBCN), and – NOX2 antibody. Representative immunoblot of one donor is shown; Lanes-1 Control; -2 Mtb+ Isotype; -3 Mtb+rIL27; -4 Mtb+aIL27; -5 Mtb+aIL10; -6 Mtb+aIL27+10. The histogram shown is for (D) N=4 donors, histograms show mean values +/- SEM. (A-C, E-F and H-I) Each dot in the plots depicts data of each individual donor, the plots include observations from 25^th^ to 75^th^ percentile. The horizontal line represents the median value. *p<0.05, **p<0.005.

Our findings so far suggest that IL-27 augments intracellular *M. tuberculosis* replication, and inhibits the anti-mycobacterial activity of macrophages. IL-27 driven inhibition of anti-mycobacterial activity is dependent on LC3B-II but does not affect autophagic flux. In the next experiments, we sought to investigate the anti-mycobacterial pathway inhibited by IL-27. It is well established that IL-27 inhibits phagosomal acidification by blocking vacuolar ATPases (V-ATPase) ^15–17^ Consistent with prior studies, we found increased expression of V-ATPase in *M. tuberculosis*-infected macrophages compared to uninfected controls (0.33± 0.2 vs 0.74 ± 0.2; p=0.02), addition of rIL-27 significantly inhibited (0.74 ± 0.2 vs 0.05 ± 0.1; p=0.007) V-ATPase expression. Additionally, we found neutralizing IL-27 increased V-ATPase expression but its level remained less than *M. tuberculosis-infected* macrophages (0.74 ± 0.2 vs 0.54± 0.2; p=0.01) (Figure 4A).

*M. tuberculosis* containing phagosomes generated via phagocytosis or autophagy eventually fuse with lysosomes. Thus, phagosomal acidification is the ultimate stage of mycobacterial degradation. Similar to autophagy, recent studies have identified LC3 associated phagocytosis (LAP) is also characterized by LC3 lipidation but is distinct from the autophagy ^29, 30^. Since we found that IL-27 inhibits LC3B lipidation but does not affect p62 expression, we sought to investigate whether IL-27 has any effect on the proteins involved in the LAP pathway in *M. tuberculosis-infected* macrophages. In contrast to autophagy, induction of LAP requires the expression of RUN-domain containing protein (RUBCN). RUBCN is essential for downstream events such as reactive oxygen species (ROS) production by nicotinamide adenine dinucleotide phosphate oxidase-2 (NOX2); thus the proteins RUBCN and NOX2 are unique to LAP. LAP mediated lysosomal-trafficking has been previously observed in cells infected with *M. tuberculosis*. Here we observed, compared to uninfected controls, an increased expression of RUBCN and NOX2 in *M. tuberculosis-infected* macrophages (-0.32 ± 0.2 vs 0.25 ± 0.1; p=0.02, and -0.08 ± 0.1 vs 0.74 ± 0.2; p=0.05, respectively) (Figures 4B and C). Addition of rIL-27 inhibited RUBCN and NOX2 expression (0.25 ± 0.1 vs -0.05 ± 0.1; p=0.03, and 0.74 ± 0.2 vs 0.03 ± 0.01; p=0.03, respectively) (Figure 4B and C, and Supplementary Figure 5A). Of note, neutralizing IL-27 increased RUBCN and NOX2 expression, but their levels remained less than *M. tuberculosis-infected* macrophages (0.25 ± 0.1 vs 0.05 ± 0.1; p=0.02, and 0.74 ± 0.2 vs 0.32 ± 0.1; p=0.2, respectively) (Figures 4B and C). Importantly ROS is critical for the anti-*M. tuberculosis* activity of macrophage. We have previously shown that IL-27 inhibits the anti-*M. tuberculosis* activity in the settings of HIV-*M. tuberculosis* co-infection. Here we investigated the effect of IL-27 in ROS generation by macrophages in response to *M. tuberculosis* infection. We found, compared to uninfected controls the expression of ROS was more in macrophages infected with *M. tuberculosis* (10192±921 vs 11366.5±1121; p=0.04). ROS expression decreased in *M. tuberculosis* infected macrophages treated with rIL-27 (11366.5±1121 vs 9055.3±385; p=0.05) (Figure 4D-F).

The Immunosuppressive cytokine IL-10 is produced by macrophages in response to *M. tuberculosis* infection and inhibits phagolysosomal mediated control of mycobacteria ^31, 32^. IL-27 modulates IL-10 function and controls TLR induced macrophage IL-10 production. We studied if neutralizing IL-27 in *M. tuberculosis*-infected macrophages regulate IL-10 production. Corroborating previous studies, the quantity of IL-10 in the culture supernatant of *M. tuberculosis*-infected macrophages was more than uninfected controls (undetectable vs 1051 ± 368 pg/ml; p= 0.05) (Figure 4G). IL-10 quantity further increased when *M. tuberculosis*-infected macrophages were cultured in the presence of neutralizing IL-27 antibody (1051 ± 368 vs 1586 ± 480 pg/ml; p= 0.05 pg/ml; p= 0.05) (Figure 4G).

Subsequently, we investigated the effect of neutralizing IL-10 on the expression of proteins unique to LAP. For this, *M. tuberculosis*-infected macrophages were cultured in the presence of rIL-27, neutralizing IL-27, neutralizing IL-10, or a combination of neutralizing IL-27 and IL-10, and the expression of RUBCN and NOX2 was determined. Consistent with Figures 5B and C, levels of RUBCN and NOX2 were more in the cellular lysates of *M. tuberculosis*-infected macrophages, which went down in the presence of rIL-27 (Figures 4H and I). Neutralizing IL-27 and IL-10 increased RUBCN expression, but its level remained less than *M. tuberculosis*-infected macrophages cultured without neutralizing antibodies. Neutralizing both IL-27 and IL-10 significantly increased RUBCN expression when compared to *M. tuberculosis*-infected macrophages cultured without neutralizing antibodies (0.44 ± 0.08 vs 1.11 ± 0.12; p=0.03) (Figure 4I). Similarly, Neutralizing IL-27 and IL-10 increased NOX2 expression, but its level remained less than *M. tuberculosis*-infected macrophages cultured without neutralizing antibodies. Of note, neutralizing both IL-27 and IL-10 significantly increased NOX2 expression when compared to *M. tuberculosis*-infected macrophages cultured without neutralizing antibodies (0.47 ± 0.08 vs 0.86 ± 0.2; p=0.05) (Figure 4H). Neutralizing both IL-27 and IL-10 also increased V-ATPase in *M. tuberculosis*-infected macrophages; however, the effect was less pronounced (Supplementary Figure 5B). Taken together, these studies establish that IL-27 inhibits the anti-mycobacterial activity of macrophages by augmenting IL-10, and prevents LAP mediated *M. tuberculosis* clearance.

## 4 Discussion

Macrophages are central to innate immune-mediated control of *M. tuberculosis*. Activation of TLR on human macrophages induces proinflammatory cytokine production, which augments lysosomal activity for bacterial clearance. Prior studies have shown that *M. tuberculosis* hijacks antimicrobial machinery of macrophages for its own survival ^33–35^. For example, following phagocytosis of *M. tuberculosis*, macrophages begin to produce various immunoregulatory cytokines that contribute to the inhibition of bacterial clearance. Recent studies have shown that the cytokine IL-27 expressed by activating myeloid cells including dendritic cells and macrophages, modulate both macrophage and T-cell activity during *M. tuberculosis* infection ^14–16^. IL-27 negatively regulates macrophage response and impedes the control of intracellular *M. tuberculosis*. In this research, we provide evidence that IL-27 inhibits hCAP and interferes with LC3 associated phagocytosis (LAP) pathway, which ultimately inhibit the expression of V-ATPase critical for bacterial clearance.

TLRs 2 and 4 are the major pathogen recognition receptors (PRR) for *M. tuberculosis*. Their ligation leads to phosphorylation and activation of transcription factor NF-κB, resulting in cytokine production ^2, 36^. The mycobacterial 19 kDa lipoprotein (LpqH) and heat shock protein 60/65 are the major ligands for TLR -2 and -4, respectively, which leads to induction of anti-mycobacterial cytokines IL-12, IL-6, IL-1β, TNFα ^33, 37, 38^. The concurrent induction of IL-10, IL-4, and TGF-β by mycobacterial molecules such as lipoarabinomannan, phosphatidylinositol mannoside, triacylated or diacylated lipoproteins inhibit IFN-γ signaling in macrophages, allowing *M. tuberculosis* to evade host immune response ^39^. In this regard, the recently identified immunoregulatory cytokine IL-27 opposes IL-12 mediated *M. tuberculosis* clearance ^16, 17^. We found IL-27 was increased in response to *M. tuberculosis* infection in our system. However, the quantity of IL-27 secreted is much less than other cytokines. We did find significant expression of IL27p28 in the cellular lysates (Supplementary Figure 2) suggesting that: (i) even a small quantity of secreted IL-27 is sufficient to transmit biological function to neighboring cells, and (ii) IL-27 retained inside macrophages inhibits anti-mycobacterial signals, impeding mycobacterial control. In mice, IL27p28 (p28) is induced both in IFN-γ independent and dependent manner by utilizing distinct pathways under the two conditions ^40–42^. IFN-γ independent p28 production is dependent on TLR-4/myeloid differentiation factor 88 (MyD88)-NF-kB c-Rel pathway; whereas in response to IFNγ, p28 is produced in an interferon regulatory factor -1 (IRF1)-dependent manner ^40^. Although, IFNγ has a profound effect on the anti-mycobacterial activity of macrophages, here we provide the evidence that *M. tuberculosis* infection can induce IL-27 expression in IFN-γ independent manner, suppressing anti-mycobacterial innate immune response. We recently reported that in HIV-*M. tuberculosis* co-infection, IL-27 is expressed by myeloid-derived suppressor cells with diminished MyD88 gene expression^19^. Here we report IL-27 expression by macrophages was inhibited by blocking TLR-2 and -4, and infection with *M. tuberculosis* strain lacking TLR ligand LpqH lipoprotein. This suggests cell type-dependent mechanism(s) for IL-27 expression. Dissecting the mechanism of IL-27 induction was beyond the scope of this research, however we are currently investigating the pathways activated by *M. tuberculosis* for IL-27 expression.

Proinflammatory cytokines are critical for innate immune-mediated control of *M. tuberculosis,* and the role of IL-27 and IL-27R in altering this response is just being defined. For example, aerosol infection of mice with *M. tuberculosis* that lack IL-27R (WSX1-KO) increases the production of TNFα and IL-12p40 ^14^. Similarly, treatment of macrophages with soluble IL27R (sIL27R) augments IL-12 induced TNF-α, IL-1β, IFN-γ, and I-TAC in response to *M. tuberculosis* infection ^17^. The findings in this study demonstrating inhibition of anti-mycobacterial cytokines TNFα, IL-1β, and IL-6 by IL-27 supports these previous studies. Additionally, our multiplex cytokine analysis identified a pronounced effect of IL-27 on IL-15 production but did not affect IL-8, -18, and -23 production. The ability of macrophages to activate the antimicrobial peptide hCAP (LL-37) pathway requires IL-15 and is an important innate host defense for *M. tuberculosis* clearance ^12, 28, 43^. Cytokines activate hCAP in a vitamin-D (vit D) dependent manner, with IL-15 iducing 25D-1α-hydroxylase (Cyp27b1) and subsequent bioconversion of circulating 25-hydroxyvitamin D3 into bioactive 1,25D_3_ leading to the induction of hCAP ^10, 27^. The commercial serum we used to culture macrophages contained natural 25(OH) D3 at a range previously shown to be sufficient to trigger hCAP expression ^27^. Therefore, the suppression of Cyp27b1 and subsequent LL-37 (Figure 3A and B) by IL-27 could be a direct effect of IL-15 inhibition by IL-27 (Figure 2I). One pathway that links LL-37 to mycobacterial degradation is the activation of autophagy, which results in autophagosome formation and their subsequent fusion with lysosomes ^9, 28^. Notably virulent *M. tuberculosis* has developed strategies that impair autophagy at the step of autophagic flux and autophagosome-lysosome fusion ^34, 35^. IFN-γ treatment of macrophages overcomes autophagy blockade in *M. tuberculosis-infected* cells ^10, 11^. Consistent with previous studies we found, *M. tuberculosis* infection enhanced LC3B lipidation but blocked autophagic flux as evident by the accumulation of p62 protein. Our findings suggest that IL-27 inhibits the generation of autophagosomes but does not affect p62 degradation. This is in contrast to the findings of Sharma *et al* demonstrating addition of exogenous IL-27 results in inhibition of autophagic flux ^44^. The dissimilarity in results could be due to the different systems used in the two studies; firstly, Sharma *et al* studied anti-*M. tuberculosis* mechanisms in response to IFN-γ, whereas we present the findings in the absence of IFN-γ. Secondly, we treated *M. tuberculosis*-infected macrophages with 15 ng /ml of IL-27, which is a more physiological concentration as opposed to Sharma *et al* where IL-27 was used at 50 ng/ml.

IL-27 signaling inhibits the phagolysosomal pathway, decreasing the expression of vacuolar H+-ATPase (V-ATPase) that are recruited to late endosomes and lysosomes, impairing the control of Gram +, Gram − bacteria^15, 16^. Consistent with the findings of Jun *et al* where IL-27 inhibited V-ATPase in response to *M bovis* BCG, we also found *M. tuberculosis-infected* human macrophages when treated with IL-27 inhibits V-ATPase and neutralizing IL-27 allowed for V-ATPase recovery ^16^. We and others have shown that IL-27 in myeloid cells signals through STAT3, a transcription factor that mediates anti-inflammatory effect ^14, 19, 45^. STAT3 inhibition of human macrophages by small-molecule niclosamide increases lysosomal acidification and its association with BCG ^46, 47^. The anti-inflammatory cytokine IL-10 also signals through STAT3 and prevents acidification of mycobacteria containing phagosomes ^31, 32, 48, 49^. Thus, both IL-27 and IL-10 operate together or in a similar manner to oppose, phagosomal acidification and anti-mycobacterial immune response of macrophages. Kalliolias *et al* showed that IL-27 priming of macrophages suppresses IL-10 gene expression and protein production in response to TLR synthetic ligands. In this study, we provide the first evidence that IL-27 produced during *M. tuberculosis* infection regulates IL-10 production, and neutralizing both of these cytokines profoundly affects the anti-mycobacterial activity of human macrophages. Future studies utilizing animal models of tuberculosis will address the effect of IL-27 neutralization on granuloma, cellular recruitment to the lung microenvironment, secondary lymph nodes, and containment of *M. tuberculosis in vivo*. Nevertheless, our studies on primary human macrophages suggest that regulating the cytokine environment is an important approach to establish the anti-mycobacterial state of macrophages.

Our results that *M. tuberculosis-infected* macrophages increase LC3B lipidation but do not degrade p62 are consistent with previous work showing *M. tuberculosis* has developed mechanisms(s) to evade autophagy-mediated control of the *M. tuberculosis* ^8, 34, 35, 50, 51^. Previous studies also establish that autophagy-mediated clearance of *M. tuberculosis* by macrophage requires IFN-γ induced activation signals ^9–11, 28, 52, 53^. During *M. tuberculosis* infection, lymphocytes are the primary cells producing IFN-γ, which activate macrophages in a paracrine manner ^54–58^. IFN-γ gene expression and protein production by lymphocytes in *M. tuberculosis*-infected mice is detectable as early as 10-days and 14-days post-infection ^59–61^. Our *in vitro* model without exogenous IFN-γ resembles an early infection time point when the effect of activation signals on macrophages generated by other immune cells is minimum. ROS produced by host enzymes is also critical for controlling *M. tuberculosis* both *in vitro*, in experimental disease models, and in patients suffering from chronic granulomatous disease ^62–66^. Although regulation of ROS production during *M. tuberculosis* infection is not well-defined, it is dependent on the multimeric enzyme NOX2 in TLR dependent manner, where NOX2 aggregates and activates oligomeric protein complexes to participate in the host immune defense ^67–69^. LC3 associated phagocytosis (LAP) is another phagolysosomal pathway that is characterized by the association of LC3 with phagosomal membrane ^30, 70^ and is active in *M. tuberculosis* infected cells ^76^. Consistent to LAP activation pathway, our immunoblotting, gene expression and microscopy studies demonstrate induction of NOX2 gp91^phox^ (Figure 4C and F, and Supplementary Figure 5A) and *p47^phox^* (Supplementary Figure 5B), suggesting that NOX2 plays an additional role by activating LAP pathway in *M. tuberculosis* infected macrophages ^30, 74, 76^. Upstream of NOX2 activity is the protein RUBCN, which is indispensable for LAP activity ^71^. RUBCN is characterized as an autophagy inhibitor and seems to be a potential reason of blockade in autophagic flux as observed in our study. The findings presented here showing the presence of LAP mediated control mechanism in *M. tuberculosis*-infected macrophages, corroborate with those of Koster *et al* demonstrating that *M. tuberculosis* mutant lacking CspA (Rv3484) protein undergoes LAP ^72^. Our ongoing studies are focused on understanding the order of events differentiating LAP and autophagy, and the immunological outcome of LAP in *M. tuberculosis*-infected macrophages.

In summary, our results extend the knowledge that IL-27 downregulates the anti-mycobacterial activity of macrophages. Furthermore, this study suggests that neutralizing both IL-27 and IL-10 better control *M. tuberculosis,* thus co-neutralization is of better therapeutic value during infection. Of note, this study supports presence of LAP mechanism in *M. tuberculosis* infected human macrophages. Our study provides a mechanistic overview of how IL-27 dampens the endogenous anti-mycobacterial activity of macrophages in the absence of macrophage activation signals. Taken together, these findings are suggestive of immunotherapeutic approaches that may involve the neutralization of IL-27 to control *M. tuberculosis*.

## Supplementary Material

Supplementary Figure 1

Supplementary Information
