# Supplementary material for: IL-27 Inhibits Anti-Mycobacterium tuberculosis Innate Immune Activity of Primary Human Macrophages
Source: Tuberculosis (Edinb). Author manuscript; Available in PMC 2023 May 10. (PMC10052773; doi:10.1016/j.tube.2023.102326)
Supplement: Supplementary Figure 1 [file EMS175072-supplement-Supplementary_Figure_1.doc]

**IL-27 Inhibits Anti- *Mycobacterium tuberculosis* Innate Immune Activity of Primary Human Macrophages**

Hailey Gollnick^2^, Jamie Barber^1^, Robert J Wilkinson^3,4^, Sandra Newton^5^, Ankita Garg^1,*^

^1^ Department of Infectious Diseases, College of Veterinary Medicine, University of Georgia, Athens, GA, USA

^2^ College of Veterinary Medicine, University of Georgia, Athens, GA, USA

3. Department of Infectious Diseases, Imperial College London, W12 0NN, United Kingdom

4. The Francis Crick Institute London NW1 1AT, United Kingdom

5. Section of Paediatric Infectious Disease, Department of Infectious Disease, Imperial College London, United Kingdom, W2 1PG

*Address correspondence to: Ankita Garg, PhD,

Department of Infectious Diseases

College of Veterinary Medicine

501 D.W. Brooks Dr

University of Georgia,

Athens, GA, 30602

USA

**Phone**: 706-542-4541

**Fax**: 706-542-5771

**E-mail**: [agarg@uga.edu](mailto:agarg@uga.edu)

Supplementary Figure 1


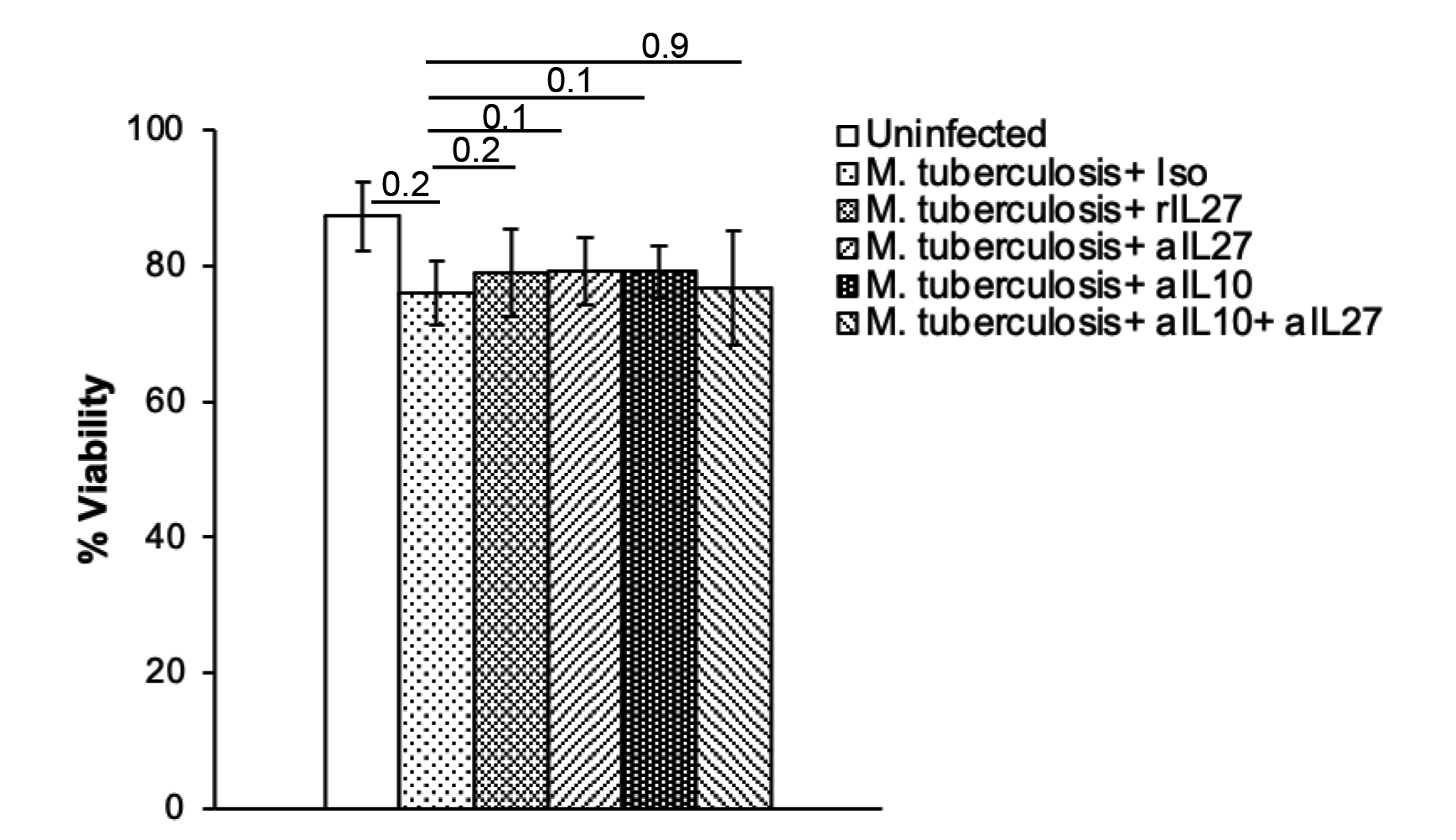


**Cellular viability of *M. tuberculosis* infected macrophages: :** Primary human macrophages were uninfected (Uninfected) or infected with *M. tuberculosis* at MOI 1:5 for 3 hours, washed with PBS to remove extracellular bacteria and treated with rIL27 (Mtb+rIL27), isotype (Mtb+Iso), neutralizing IL-27 (Mtb+aIL27), neutralizing IL-10 (Mtb+ aIL10) or neutralizing IL-27 + IL-10 (Mtb+aIL27+a10) antibodies. The quantity of lactate dehydrogenase was measured in the culture supernatant, and percentage viability calculated as in Methods. The histogram shown is for N=4 donors. Histograms show mean values +/- SEM.


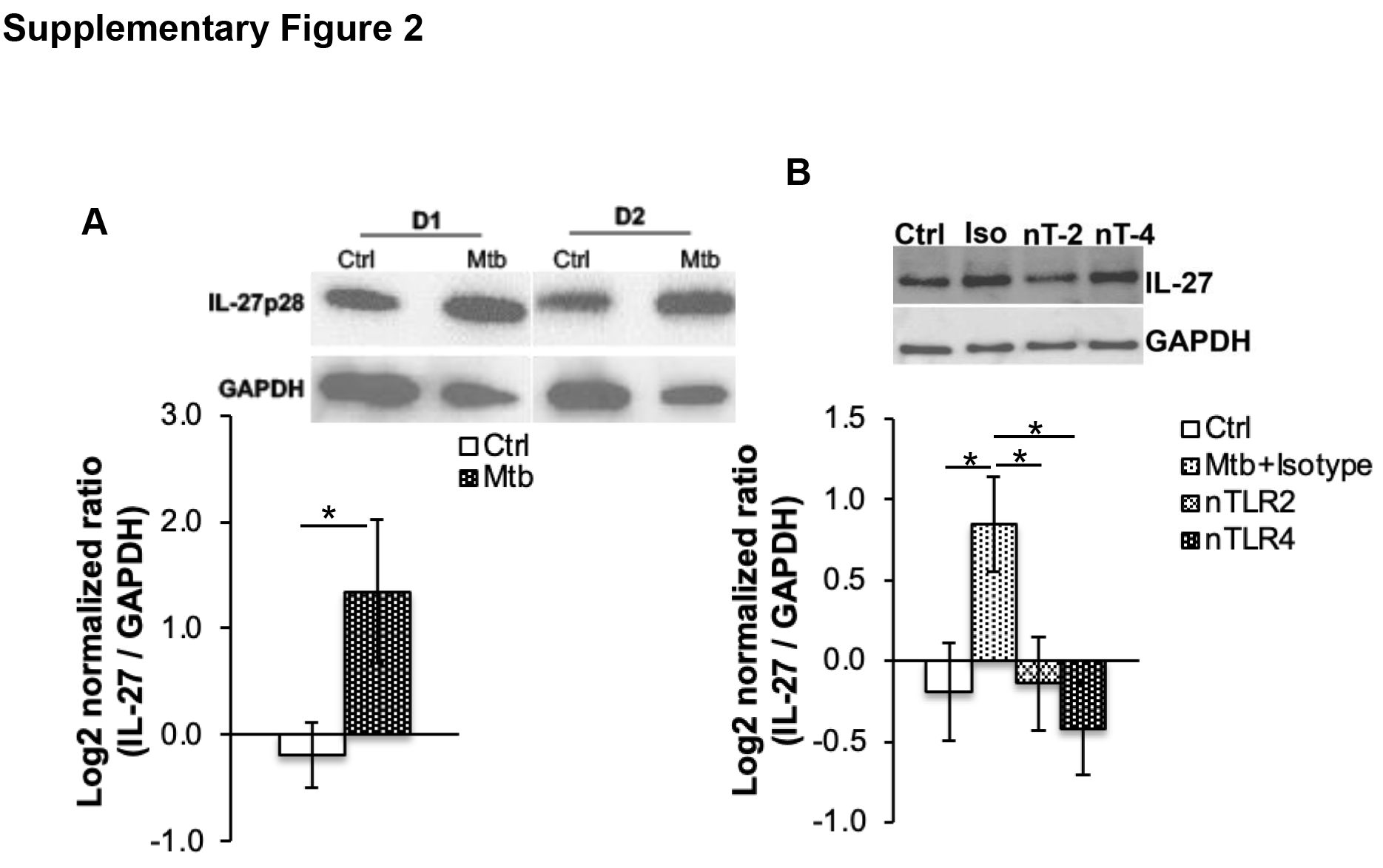
Supplementary Figure 2

**IL-27 is expressed by *M tuberculosis*-infected macrophages in TLR dependent manner:** Primary human macrophages were uninfected (Ctrl) or infected with *M. tuberculosis* (Mtb) at MOI of 1:5 for 3 hours, washed with PBS to remove extracellular bacteria **(A)** Cellular lysates were prepared at 24-48 hours post-infection and immunoblotted using anti- GAPDH and - IL27p28 antibodies. A representative immunoblot of two donors (D1 and D2) is shown. **(B)** Cells were incubated with isotype (Mtb+Isotype), blocking TLR-2 (nTLR2) or blocking TLR-4 (nTLR4) antibodies before infection with *M. tuberculosis*. Cellular lysates were prepared at 24-48 hours post-infection and immunoblotted using anti- GAPDH and - IL27p28 antibodies. A representative immunoblot of one donor is shown. The histogram shown is for N=4 donors. All histograms show mean values +/- SEM. *p<0.05.

Supplementary Figure 3


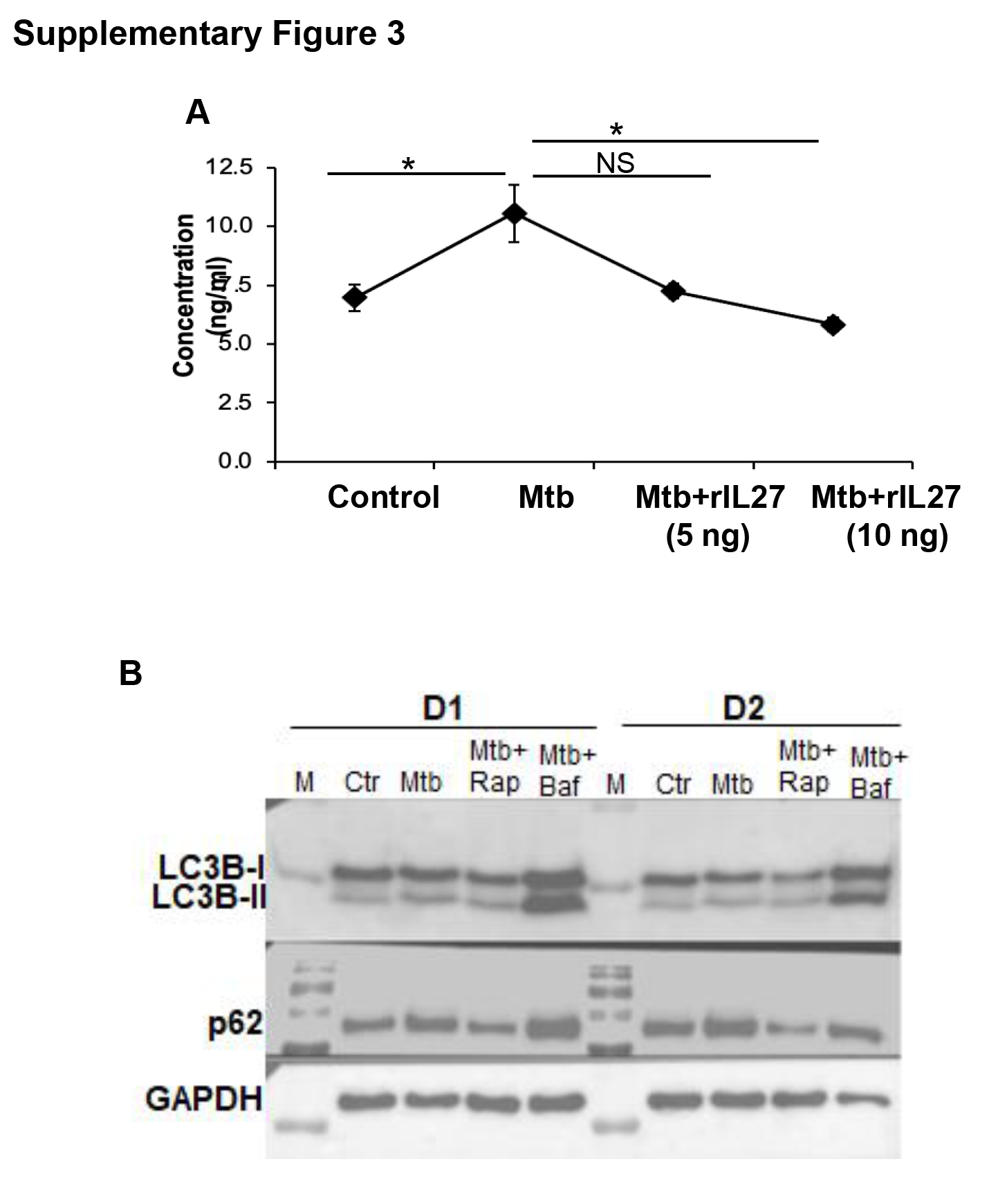


**Cathelicidin (hCAP-LL37) production and autophagy:** Primary human macrophages were uninfected (Ctrl) or infected with *M. tuberculosis* (Mtb) at MOI of 1:5 for 3 hours, washed with PBS to remove extracellular bacteria. **(A)** *M. tuberculosis*-infected cells were treated with rIL27 at the indicated concentration. The amount of hCAL LL-37 in the culture supernatants of uninfected (Control), infected and rIL27 treated cells was measured at 24-48 hours post-infection. **(B)** Total cellular lysates were prepared and immunoblotted using anti- GAPDH, - LC3B and – p62 antibody. Representative immunoblots of two donors (D1 and D2) are shown shown. M: molecular weight marker; Ctrl: control; Mtb: *M. tuberculosis*; Rap: Rapamycin; Baf: Bafilomycin A. (A) Data shown are for N=3 donors; mean values +/- SEM are shown. *p<0.05, NS: Non-significant


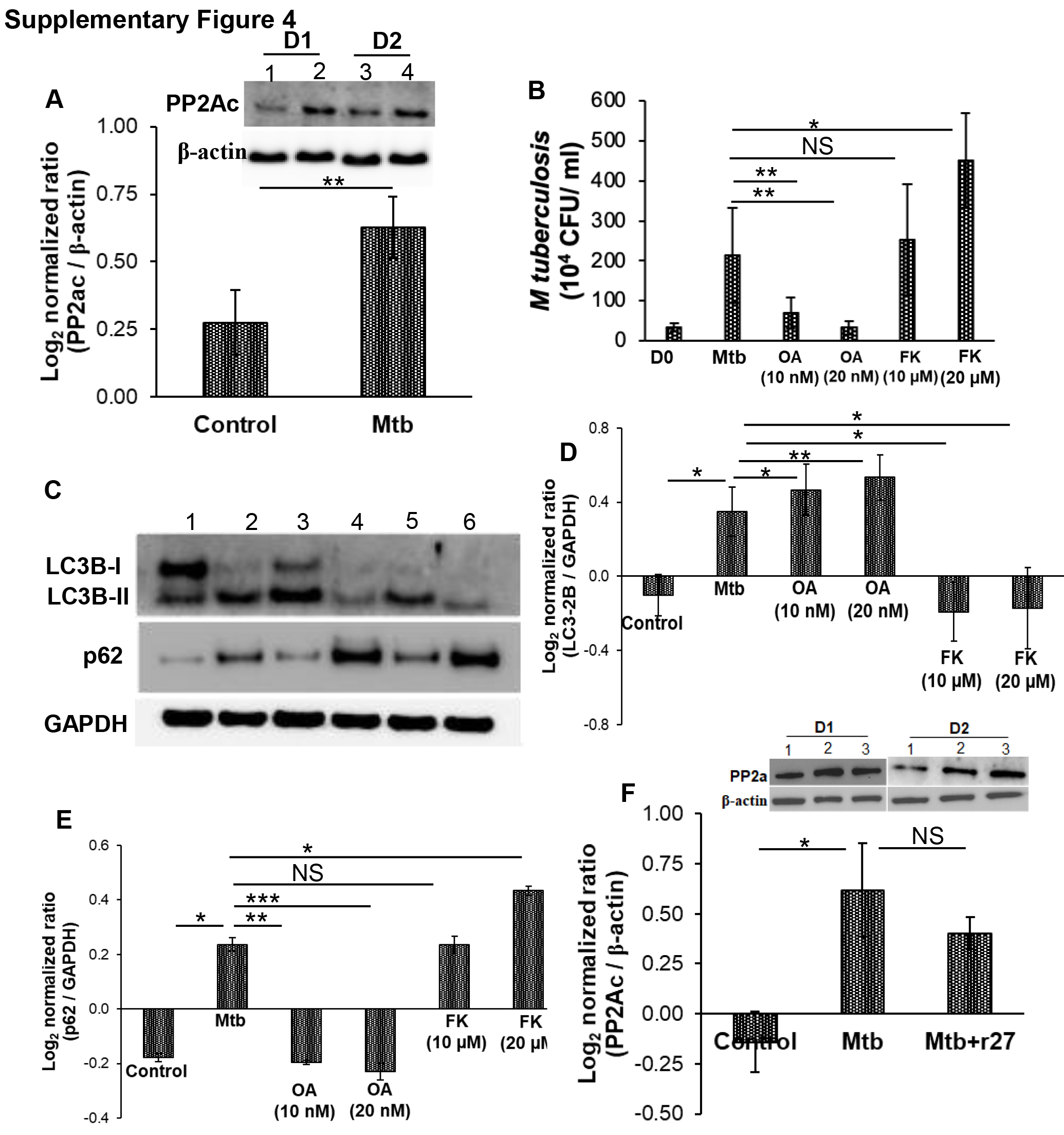


**Effect of PP2Ac on the anti-mycobacterial activity of macrophages:** Cells were uninfected (Control) or infected with *M. tuberculosis* (Mtb) at an MOI of 1:5 for 3 hours, washed with PBS to remove extracellular bacteria. **(A)** Total cellular lysates were prepared and immunoblotted using anti- β-actin and – PP2Ac antibodies. Representative immunoblot of two donors (D1 and D2) is shown; lanes 1 and 3 Control; lanes 2 and 4 Mtb infected **(B)** *M. tuberculosis* growth (colony forming units (CFU) / ml) was determined in the cellular lysates at day-0 post-infection. Infected cells were treated with vehicle (Mtb), PP2Ac antagonist (OA), or PP2Ac agonist (FK) at indicated concentration; CFU/ml was determined at day-3 post-infection. **(C-D)** Cellular lysates of uninfected (Control) and infected cells treated with vehicle (Mtb), PP2Ac antagonist (OA) or agonist (FK) were prepared and immunoblotted using anti- - GAPDH, - LC3B, and – p62 antibody. Representative immunoblot of one donor is shown; Lanes- 1 Control; -2 Mtb; -3 Mtb+OA (10 nM); -4 Mtb+OA (20 nM); -5 Mtb+FK (10 µM); -6 Mtb+FK (20 µM). **(F)** *M. tuberculosis*-infected cells were untreated (Mtb) or treated with rIL27 (Mtb+rIL27); cellular lysates of uninfected (Control) and infected cells were prepared and immunoblotted using anti- β-actin and – PP2Ac antibody. Representative immunoblot of two donors (D1 and D2) is shown; lanes- 1 control; - 2 Mtb; -3 Mtb + rIL27. The histogram shown is for (A) N=6 donors, (B) N=4 donors, (D and E) N=3 donors, (F) N=6 donors. All histograms show mean values +/- SEM. *p<0.05, **p<0.005, ***p<0.0005, NS: Non-significant.

Supplementary Figure 5


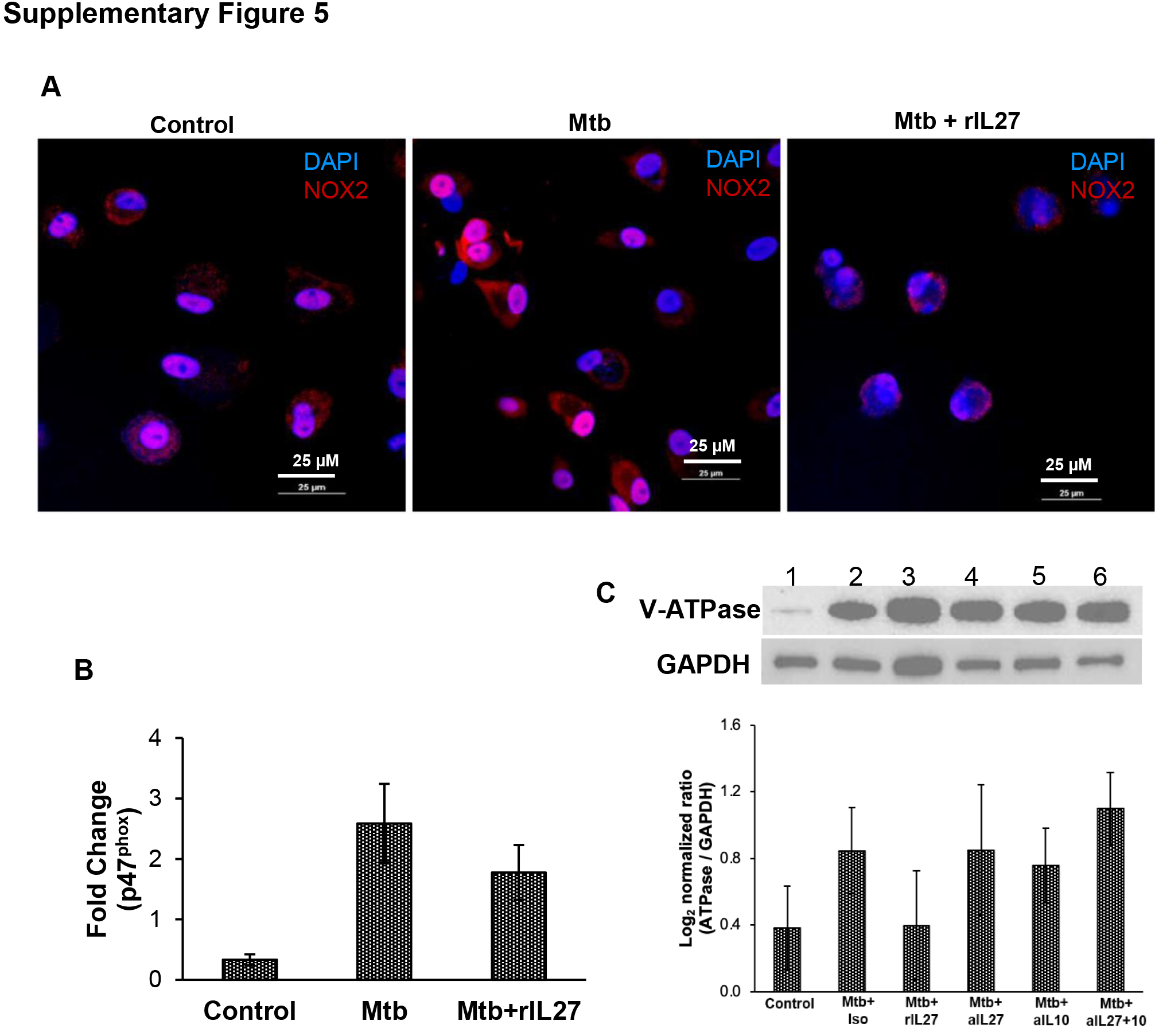


**Effect of IL-27 and IL-10 on vacuolar ATPase:** Cells were uninfected (Ctrl) of infected with *M. tuberculosis* at MOI 1:5 for 3 hours, washed with PBS to remove extracellular bacteria and cultured in the presence or absence of indicated treatment **(A)** IF was performed with anti-NOX2 (red) antibody, and nuclei were stained with DAPI (blue). **(B)** Expression of housekeeping gene HuPO and p47^phox^ was determined using SYBR green, and fold change was calculated **(C)** Cellular lysates were prepared at 24-48 hours post infection and immunoblotted using anti- GAPDH, -vacuolar ATPase (V-ATPase). Representative immunoblot of one donor is shown. Lanes- 1 Control; -2 Mtb+ Isotype; -3 Mtb+rIL27; -4 Mtb+aIL27; -5 Mtb+aIL10; -6 Mtb+aIL27+10. Histogram shown is for N=3 donors. Histogram show mean values +/- SEM. (A) Image is representative of N=3 donors.
