# Supplementary material for: IL-27 Inhibits Anti-Mycobacterium tuberculosis Innate Immune Activity of Primary Human Macrophages
Source: Tuberculosis (Edinb). Author manuscript; Available in PMC 2023 May 10. (PMC10052773; doi:10.1016/j.tube.2023.102326)
Supplement: Supplementary Information [file EMS175072-supplement-Supplementary_Information.pdf]

College of Veterinary Medicine
Department of Infectious Diseases

9th, February 2023

TO: Editor Tuberculosis

RE: Revision: Manuscript "IL-27 Inhibits Anti-*Mycobacterium tuberculosis* Innate Immune Activity of Primary Human Macrophages"

We thank the reviewers for the helpful comments and suggestions and their positive statements regarding the overall study plan and ongoing studies. We thank you for giving us an additional opportunity to revise our manuscript.

The manuscript entitled: "IL-27 Inhibits Anti-*Mycobacterium tuberculosis* Innate Immune Activity of Primary Human Macrophages" has been revised as recommended by Reviewer# 1. An item-by-item response to the Reviewer's comments follows below:

- *The abstract does not read as clearly as other sections of the manuscript and could be improved for clarity and focus.*

We have edited the Abstract and is more focused on the study now.

- *The findings with regards to autophagic flux are hard to follow as currently presented. Initially it is stated that Mtb inhibited flux, as measured by p62 expression (line 233), but then later on the same page it is stated that Mtb did not lower p62 levels (line 245).*

Thank you for noting this. Mtb inhibits flux, this is observed by increased expression of p62. Both line 233 and line 245 in the original submission mean the same observation. We have now rephrased the statement to give better clarity.

- *The authors state in the discussion that they are currently investigating the pathways through which TLR-2 and -4 engagement by Mtb may lead to induction of IL-27. This is of great interest as this is an important step to understand for any potential host-directed therapies to modulate the response to Mtb infection.*

Thank you for appreciating the ongoing studies. Hopefully, these findings will be communicated soon as a new manuscript.

- *The discussion is a bit unwieldy in parts and might read more easily if the scope were tightened (e.g., the introduction of vitamin-D dependent mechanisms and mention of populations with higher rates of vitamin-D deficiency).*

We have now edited the Discussion as suggested.

We hope you will find this manuscript suitable for publication in Tuberculosis. Thank you for your consideration.

Sincerely,

Ankita Garg, PhD
Assistant Professor

Telephone (706) 542-3473 • Fax (706) 542-5771 • Athens, GA 30602-7387 • website: www.vet.uga.edu/ID
An Equal Opportunity/Affirmative Action Institution

Click here to access/download
Submission checklist
TUBE_Checklist_for_initial_submissions_14012021_BDR.pdf
